# Supplementary material for: Surface Environment and Energy Density Effects on the Detection and Disinfection of Microorganisms Using a Portable Instrument
Source: Sensors (Basel). 2023 Feb 14;23(4):2135. doi: 10.3390/s23042135 (PMC9968048; doi:10.3390/s23042135)
Supplement: Supplementary file 1 [file sensors-23-02135-s001.zip › sensors-2201890-supplementary.pdf]

sensors

Article

Surface Environment and Energy Density Effects on the Detection and Disinfection of Microorganisms Using a Portable Instrument

Sungho Shin [1], Brianna Dowden [1], Iyll-Joon Doh [1], Bartek Rajwa [2], Euiwon Bae [3] and J. Paul Robinson [1,4,*]

[1] Department of Basic Medical Sciences, Purdue University, West Lafayette, IN 47907, USA; shin331@purdue.edu (S.S.); dowdenb@purdue.edu (B.D.); idoh@purdue.edu (I.-J.D.)
[2] Bindley Bioscience Center, Purdue University, West Lafayette, IN 47907, USA; brajwa@purdue.edu
[3] School of Mechanical Engineering, Purdue University, West Lafayette, IN 47907, USA; ebae@purdue.edu
[4] Weldon School of Biomedical Engineering, Purdue University, West Lafayette, IN 47907, USA
* Correspondence: jpr@cyto.purdue.edu

Supplementary Materials

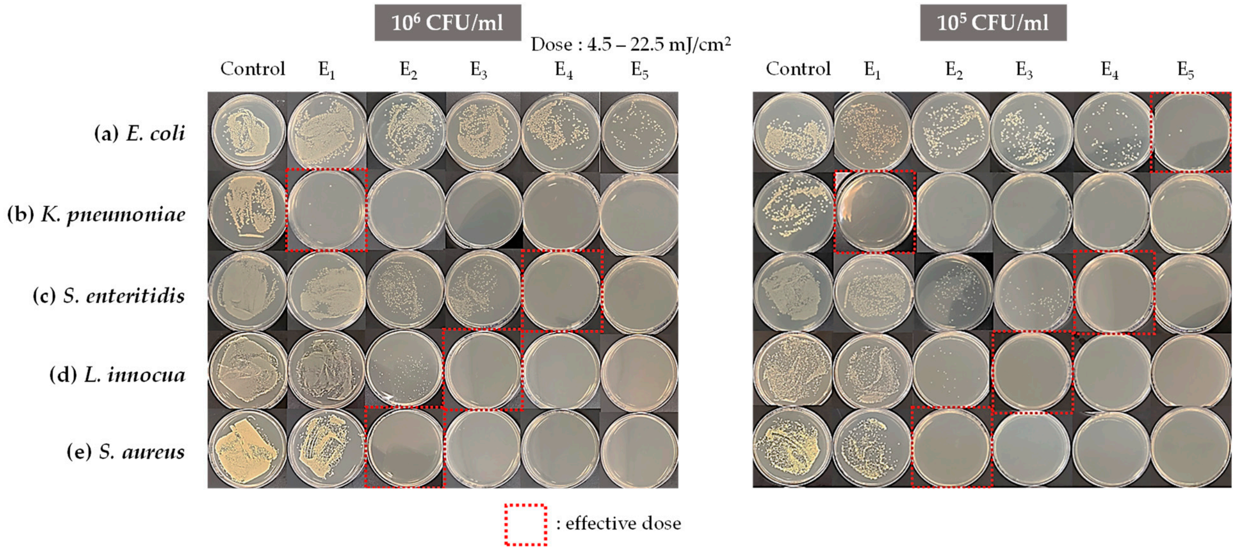

Figure S1. Plate pictures after incubation at two different concentrations (10^6 and 10^5 CFU/ml) for (a) *E. coli*, (b) *K. pneumoniae*, (c) *S. enteritidis*, (d) *L. innocua*, and (e) *S. aureus*.
